# Hematomas in musculoskeletal tissue donation: do they really increase microbiological risk?

**DOI:** 10.1007/s10561-026-10224-4

**Published:** 2026-05-26

**Authors:** Rossella Ravaglia, Andrea Della Valle, Francesco Puglia, Jacopo Menini, Alessandra Menon, Riccardo Compagnoni, Pietro Simone Randelli

**Affiliations:** 1 Scuola di specializzazione di Ortopedia e Traumatologia, Università degli Studi di Milano, Milan, Italy; 2 U.O.C. Ortopedia e Traumatologia Pediatrica, ASST G. Pini-CTO, Milan, Italy; 3 U.O.C. Patologie Vertebrali e Scoliosi, ASST G. Pini-CTO, Milan, Italy; 4 U.O.C. 1° Clinica Ortopedica, ASST G. Pini-CTO, Milan, Italy; 5 Department of Biomedical, Surgical and Dental Sciences, Università degli Studi di Milano, Via della Commenda, 10, 20122 Milan, Italy; 6 Laboratory of Applied Biomechanics, Department of Biomedical Sciences for Health, Università degli Studi di Milano, Via Mangiagalli 31, 20133 Milan, Italy; 7 Research Center for Adult and Pediatric Rheumatic Diseases (RECAP-RD), Department of Biomedical Sciences for Health, Università degli Studi di Milano, Via Mangiagalli 31, 20133 Milan, Italy

**Keywords:** Hematoma, Musculoskeletal tissues, Tissue banking, Microbiological contamination, Allografts, Cadaveric donors

## Abstract

Hematoma presence in musculoskeletal tissues procured from cadaveric donors is traditionally considered a potential risk factor for microbiological contamination. This perception has led to the frequent exclusion of hematoma-bearing tissues from allograft use, despite limited scientific evidence supporting this practice. We conducted a prospective case–control study on 234 musculoskeletal tissues retrieved from 16 cadaveric donors between September 2023 and December 2024. For each tissue, data on hematoma presence and microbiological culture results were collected. Fisher’s exact test was used to assess the association between hematoma presence and microbiological positivity. Odds ratios (OR) and 95% confidence intervals (CI) were calculated. A secondary intra-donor analysis examined concordance of microbiological results in paired bilateral tissues. Hematomas were observed in 25 tissues (10.7%). Overall microbiological positivity was 5.1% (12/234), with 8.0% (2/25) in tissues with hematoma and 4.8% (10/209) in tissues without hematoma. The difference was not statistically significant (OR 1.73; 95% CI: 0.35–8.48; *p* = 0.62). No bilateral concordance of positivity was observed in paired tissues, and unilateral positivity was found only in tissues without hematoma. Hematoma presence in musculoskeletal tissues from cadaveric donors does not appear to significantly increase microbiological contamination risk. These findings suggest that hematoma alone should not be considered an automatic exclusion criterion. Larger studies are needed to confirm these results and guide evidence-based updates to tissue bank policies.

## Introduction

The presence of hematoma near musculoskeletal tissues at the time of procurement is traditionally considered a potential risk factor for microbiological contamination and is often used as an exclusion criterion for transplantation. However, this practice is based more on prudential assumptions than on solid scientific evidence or formal regulatory directives. Currently, major international guidelines for tissue banks, such as those of the American Association of Tissue Banks (AATB) and the EU Tissue and Cells Directives, do not explicitly mandate the automatic exclusion of tissues with hematoma, instead recommending case-by-case evaluation based on microbiological quality and safety (American Association of Tissue Banks 2022; European Union 2004).

In recent years, musculoskeletal tissue banks have played an increasingly central role in ensuring the safety and quality of donated tissues. Data from the Regional Musculoskeletal Tissue Bank of Lombardy have shown a steady increase in the use of allografts in orthopedic surgery and a progressive reduction in microbiological positivity rates, attributed to improved procurement techniques and the training of dedicated teams (Della Valle et al. 2024). The resilience and adaptability of regional musculoskeletal tissue banks, particularly during the COVID-19 pandemic, have been highlighted in recent literature, underscoring their capacity to ensure safe tissue procurement under challenging conditions (Puglia et al. 2025).

In the clinical setting, hematoma formation has been associated with a potential increase in infection risk, particularly in postoperative contexts and in frail patients (Charles et al. 2023; Cheung et al. 2008; Murray et al. 2012; Rogani et al. 2020). This increased susceptibility to infection has been attributed to the hematoma microenvironment, which may impair local immune responses, alter macrophage activity, and create a nutrient-rich substrate conducive to bacterial proliferation (Chaudry and Ayala 1993). However, the specific impact of hematoma presence on allogeneic tissues procured from cadaveric donors remains poorly defined.

Given the growing demand for musculoskeletal allografts and the need to minimize discard rates due to unsubstantiated exclusion criteria, the primary aim of this study was to assess whether the presence of hematoma in procured musculoskeletal tissues is associated with an increased risk of microbiological positivity. As a secondary objective, we investigated the concordance of microbiological results and hematoma presence in paired bilateral tissues from the same donor, to explore possible intra-donor patterns of contamination.

We hypothesized that hematoma presence is not associated with microbiological contamination and that any positivity would likely result from systemic donor factors or procedural variables during procurement.

## Materials and methods

This was a prospective study conducted at the Regional Musculoskeletal Tissue Bank, covering all musculoskeletal tissue procurements performed from September 2023 to December 2024. A total of 16 cadaveric donors were included, yielding 234 musculoskeletal tissues for analysis, including both bone and tendon tissues. All donors were classified as DBD (donation after brain death, heart‑beating donors). This classification is consistent with standard definitions in transplantation research, where DBD refers to brain‑dead donors maintained on circulatory support until tissue procurement (Ortas et al. 2025).

All donors met eligibility criteria for tissue donation and were processed according to standard operating procedures of the tissue bank. Tissue procurement was performed only after consent for donation had been obtained according to applicable regulations and tissue bank procedures.

For each tissue, data collected included donor demographic information (age, sex), tissue category (bone or tendon), specific tissue retrieved, presence or absence of visible hematoma at the time of procurement, and microbiological culture results.

Tissues were classified as “hematoma positive” if a hematoma was visually detected during procurement by the surgical team.

Microbiological testing was performed according to the standardized operating procedures adopted by the tissue bank, using dedicated personnel trained in aseptic techniques. For each tissue, microbiological sampling was carried out before packaging and freezing and included both a swab sample and a tissue sample for aerobic, anaerobic, and fungal investigation. Swab specimens were collected using the eSwab system, while tissue samples were processed in THIOL enrichment broth. Culture processing was performed by automated plating procedures. Microbiological samples were sent immediately at room temperature to the microbiology laboratory. In contrast, procured tissues were transported at − 80 °C in dry ice containers and stored in a dedicated quarantine freezer at − 80 °C until microbiological results became available. Cultures were considered positive if any microbial growth was detected during the standard incubation period.

Statistical analysis was performed using R version 4.2.2 (R Foundation for Statistical Computing, Vienna, Austria). The association between hematoma presence and microbiological positivity was evaluated using Fisher’s exact test. Odds ratios (OR) with 95% confidence intervals (CI) were calculated. A *p*-value < 0.05 was considered statistically significant.

For each donor, the length of hospital stay before procurement was also recorded and explored as a donor-level variable. Differences between donors with at least one positive tissue culture and those without positive cultures were assessed using the Mann–Whitney test.

A secondary intra-donor analysis was performed to compare microbiological results between paired bilateral tissues (e.g., right and left tibia) from the same donor. The aim was to assess whether the presence of hematoma or microbiological positivity showed intra-donor symmetry.

All procedures complied with the Declaration of Helsinki and the General Data Protection Regulation (GDPR) guidelines. The study protocol was approved on May 26, 2023 by the Technical Scientific Committee (CTS) of ASST Gaetano Pini-CTO, Milan, Italy.

## Results

A total of 234 musculoskeletal tissues, including both bone and tendon tissue, were procured from 16 cadaveric donors between September 2023 and December 2024. Of these, 25 tissues (10.7%) presented a visible hematoma at the time of procurement.

The overall rate of microbiological positivity was 5.1% (12/234). Overall, 8 of 16 donations included at least one tissue with a positive culture.

Among tissues without hematoma (n = 209), 10 tested positive (4.8%). Among tissues with hematoma (n = 25), 2 tested positive (8.0%). The difference in microbiological positivity between tissues with and without hematoma was not statistically significant (odds ratio [OR]: 1.73; 95% confidence interval [CI]: 0.35–8.48; *p* = 0.62, Fisher’s exact test).

Among tissues with hematoma, the two positive cultures yielded *Staphylococcus epidermidis* and *Propionibacterium acnes*. Among tissues without hematoma, the isolated microorganisms were *Staphylococcus epidermidis* (n = 3), *Staphylococcus capitis* (n = 3), *Staphylococcus hominis* (n = 2), *Corynebacterium* species (n = 1), and *Bacillus* species (n = 1). Overall, the microbiological profile was predominantly Gram-positive.

Positive cultures were observed in both bone and tendon tissues, without clear clustering in a single tissue category.

Among donors with more than one positive tissue, no consistent intra-donor pattern was observed with respect to either the isolated microorganism or the anatomical proximity of the positive tissues.

Donor hospital stay before procurement was available for 14 of the 16 donors. No significant difference was observed between donors with at least one positive tissue culture and those without positive cultures (median 4 vs 4 days; Mann–Whitney test, *p* = 0.95).

A secondary intra-donor analysis was performed on paired bilateral tissues (e.g., right and left tibiae) from the same donor. No cases of bilateral microbiological positivity were observed. In all instances of unilateral positivity, the positive tissue did not present a hematoma, suggesting a localized rather than systemic contamination pattern.

Table 1 summarizes the distribution of microbiological positivity according to hematoma status.

**Table 1:** Microbiological positivity in tissues with and without hematoma

	Total tissues	Positive cultures (n)	Positive cultures (%)
Hematoma Absent	209	10	4.8%
Hematoma Present	25	2	8.0%
Total	234	12	5.1%

## Discussion

This study aimed to investigate whether the presence of hematoma in musculoskeletal tissues procured from cadaveric donors is associated with an increased risk of microbiological contamination. In our cohort of 234 tissues from 16 donors, we found no statistically significant association between hematoma presence and microbiological positivity (OR 1.73; *p* = 0.62). Although there was a slightly higher percentage of positive cultures in tissues with hematoma (8.0%) compared to those without (4.8%), this difference did not reach significance.

Our findings challenge the common perception that hematoma is a direct risk factor for contamination and call into question its use as an absolute exclusion criterion during tissue procurement. Current guidelines from the American Association of Tissue Banks (AATB) and the EU Tissue and Cells Directives do not specifically mandate the exclusion of tissues with hematoma, recommending instead a case-by-case assessment based on microbiological quality and safety (American Association of Tissue Banks 2022; European Union 2004).

Previous studies in surgical settings have demonstrated an association between hematoma formation and postoperative infection risk. Cheung et al. reported that hematomas requiring surgical evacuation after shoulder arthroplasty were often linked to positive cultures and deep infections (Cheung et al. 2008). Rogani et al. further illustrated the vulnerability of tissues with spontaneous muscle hematomas in frail COVID-19 patients (Rogani et al. 2020).

Charles et al. highlighted that delayed surgical drainage of hematomas beyond 72 h significantly increased infection rates in joint arthroplasty patients (Charles et al. 2023), supporting the concept that prolonged hematoma presence in vivo may favor bacterial growth. However, these clinical findings may not directly translate to tissue banking.

Della Valle et al. reported a progressive reduction in microbiological contamination rates over an 11-year period in their regional tissue bank, attributing this to improved procurement techniques and dedicated training of personnel (Della Valle et al. 2024). These improvements may mitigate potential risks associated with hematomas in cadaveric tissues.

A similar experience was reported by Azkia et al., who described how a local, non-commercial tissue bank connected to an organ donor program was able to maintain high-quality standards at very low costs over a ten-year period (Azkia et al. 2025).

In our secondary intra-donor analysis, no cases of bilateral microbiological positivity were observed, and unilateral positivity was always found in tissues without hematoma.

This suggests that contamination is more likely a localized event, possibly related to procurement handling, rather than a systemic issue affecting all tissues from a single donor. Similar findings were reported by Viñuela-Prieto et al., who found that contamination often correlated with specific procurement factors such as extraction time and number of samples, rather than donor-related factors (Viñuela-Prieto et al. 2019).

The microbiological profile of positive cultures was uniformly Gram-positive, with no clear pattern specifically associated with hematoma presence.

This study has several limitations. First, the sample size is relatively small, which may limit the statistical power to detect subtle associations. Second, data on the size and age of hematomas were not collected, which could influence their potential for bacterial colonization. Future prospective studies should include a standardized classification of hematoma size and age to better define their clinical relevance in tissue procurement.

## Conclusion

The presence of hematoma in musculoskeletal tissues procured from cadaveric donors does not appear to be significantly associated with microbiological contamination. Although a non-significant trend toward higher positivity was observed in tissues with hematoma, our findings suggest that hematoma alone should not constitute an absolute exclusion criterion.

Larger prospective studies incorporating a standardized characterization of hematomas are needed before firm recommendations can be made or tissue bank guidelines refined.

## Data Availability

The datasets generated and analyzed during the current study are available from the corresponding author on reasonable request.
